# RasGRP1, but Not RasGRP3, Is Required for Efficient Thymic β-Selection and ERK Activation Downstream of CXCR4

**DOI:** 10.1371/journal.pone.0053300

**Published:** 2013-01-07

**Authors:** Dominic P. Golec, Nancy A. Dower, James C. Stone, Troy A. Baldwin

**Affiliations:** 1 Department of Medical Microbiology and Immunology, University of Alberta, Edmonton, Alberta, Canada; 2 Department of Biochemistry, University of Alberta, Edmonton, Alberta, Canada; 3 Department of Pediatrics, University of Alberta, Edmonton, Alberta, Canada; Institut Jacques Monod, France

## Abstract

T cell development is a highly dynamic process that is driven by interactions between developing thymocytes and the thymic microenvironment. Upon entering the thymus, the earliest thymic progenitors, called CD4^−^CD8^−^ ‘double negative’ (DN) thymocytes, pass through a checkpoint termed “β-selection” before maturing into CD4^+^CD8^+^ ‘double positive’ (DP) thymocytes. β-selection is an important developmental checkpoint during thymopoiesis where developing DN thymocytes that successfully express the pre-T cell receptor (TCR) undergo extensive proliferation and differentiation towards the DP stage. Signals transduced through the pre-TCR, chemokine receptor CXCR4 and Notch are thought to drive β-selection. Additionally, it has long been known that ERK is activated during β-selection; however the pathways regulating ERK activation remain unknown. Here, we performed a detailed analysis of the β-selection events in mice lacking RasGRP1, RasGRP3 and RasGRP1 and 3. We report that RasGRP1 KO and RasGRP1/3 DKO deficient thymi show a partial developmental block at the early DN3 stage of development. Furthermore, DN3 thymocytes from RasGRP1 and RasGRP1/3 double knock-out thymi show significantly reduced proliferation, despite expression of the TCRβ chain. As a result of impaired β-selection, the pool of TCRβ^+^ DN4 is significantly diminished, resulting in inefficient DN to DP development. Also, we report that RasGRP1 is required for ERK activation downstream of CXCR4 signaling, which we hypothesize represents a potential mechanism of RasGRP1 regulation of β-selection. Our results demonstrate that RasGRP1 is an important regulator of proliferation and differentiation at the β-selection checkpoint and functions downstream of CXCR4 to activate the Ras/MAPK pathway.

## Introduction

Hematopoietic progenitor cells enter the thymus from the bone marrow where they undergo a dynamic and highly regulated process of differentiation that culminates with the export of mature T cells. The differentiation of progenitors is controlled by interactions between the progenitor and thymic stromal cells that ultimately activate various signal transduction pathways [1]. These signal transduction pathways regulate the expression of key transcription factors that are required for differentiation. One of the key signaling pathways that is activated at various stages of intrathymic T cell development is the canonical Ras/Erk pathway.

The progenitors that seed the thymus initially lack expression of the CD4 and CD8 T cell co-receptors and are termed ‘double negative’ (DN). DN thymocytes are a heterogeneous population that can be further sub-divided based upon the expression of various cell surface molecules including CD44 and CD25. DN1 thymocytes are CD44^+^CD25^−^ with upregulation of CD25 marking entry into the DN2 stage. It is within the DN2 stage that TCRβ, γ and δ gene loci begin rearrangement with completion of TCRβ rearrangement at the CD44^−^CD25^+^ DN3 stage. Pairing of TCRβ with pre-Tα produces the pre-TCR that signals the DN3 thymocytes to undergo a process termed ‘β-selection’. The characteristic features of β-selection include commitment to the αβ-T cell lineage, continued differentiation, proliferation, survival and cessation of recombination at the TCRβ locus. Thymocytes that pass the β-selection checkpoint enter the CD44^−^CD25^−^ DN4 stage before CD4 and CD8 are upregulated to generate ‘double positive’ (DP) thymocytes [2]. Following a productive TCRα rearrangement and pairing with TCRβ to produce a mature αβTCR, the DP thymocyte is then subjected to positive and negative selection based upon the specificity of the mature TCR for self-peptide MHC complexes [3].

Currently it is thought that signals downstream of the pre-TCR, Notch and CXCR4 drive DN3 thymocytes through the β-selection checkpoint [4]. The signal transduction pathway downstream of the pre-TCR is thought to be similar to that of the mature αβ-TCR. For example, mice deficient in Zap70 and Syk, LAT or SLP-76 show a profound block in T cell development at the β-selection checkpoint [5]–[9]. Additionally, it is known that signals downstream of the pre-TCR can activate the canonical Ras/Erk pathway [10]. While Notch signaling is critically important at earlier stages of DN thymocyte development (for recent review see [11]), at the β-selection checkpoint, Notch appears to cooperate with pre-TCR signals to promote survival and metabolic activity through the PI3K pathway. It was recently demonstrated that the chemokine receptor CXCR4 is required as a ‘co-stimulatory’ receptor for β-selection [12], [13]. Again, the pre-TCR appears to cooperate with CXCR4 possibly through a PI3K mediated program of survival.

While it is known that the Ras/Erk pathway is activated during β-selection, the identity of the upstream Ras activator was unclear. Ras is a small, lipidated G-protein whose activity is regulated by guanine nucleotide binding. Ras is allosterically activated by GTP binding while the GDP bound form is inactive. Guanine nucleotide exchange factors (GEFs) catalyse the exchange of GDP for GTP thereby activating Ras, while an intrinsic GTPase activity is enhanced by GTPase activating proteins (GAPs). Ras activation leads to the recruitment and activation of the Raf kinase that can phosphorylate and activate MEK that in turn phosphorylates and activates Erk1 and 2. Erk1 and 2 can then modulate gene expression that influences many cellular processes including proliferation, survival and lineage commitment to name but a few [14]. In lymphocytes, there are two major families of GEFs that regulate Ras activity: the son of sevenless (Sos) and Ras guanylnucleotide releasing protein (RasGRP) families. The RasGRP family consists of four members (RasGRP1–4). RasGRP1 is expressed mainly in T and B cells while RasGRP3 is prominently expressed in B cells. Both RasGRP1 and 3 are regulated by the binding of their C1 domain to diacylglycerol and also have the potential to be regulated by PKC phosphorylation [15]–[18]. The role of other domains in the regulation of RasGRP activity is currently unclear. There are two Sos family members, Sos1 and Sos2. The Sos family is also regulated by recruitment to membranes through interactions with adaptor molecules such as Grb-2 and Shc. Additionally, it has recently been demonstrated that allosteric, or GTP-bound, Ras binding as well as the binding of PIP2 to the DH-PH domain of Sos increases the activity of Sos likely through release of the autoinhibitory domain [19]. Interestingly, one model of Ras activation in T cells suggests that RasGRP1 and Sos operate in a feedforward loop where initial RasGRP1-mediated Ras activation potentiates Sos activation resulting in an analogue to digital conversion of Ras activity [20], [21]. The relative roles of the RasGRP and Sos families in Ras activation during the various stages of T cell development and activation has been controversial, however, recent work has begun to resolve this issue. In a seminal paper by Dower et al., RasGRP1 was shown to be required for thymocyte positive selection [22]. Furthermore, it was recently demonstrated that RasGRP1-mediated Ras activation was required for invariant natural killer T cell (iNKT) development [23]. However, while the Ras/Erk pathway was known to be activated at the β-selection checkpoint, until recently, a role for the RasGRP family at this stage of thymocyte development had not been described. In a recent report by the Zhang group, RasGRP1 knock out (KO) mice were found to display a partial block in β-selection that was augmented by simultaneous elimination of RasGRP4. RasGRP4 KO mice did not display any impairment in β-selection [24]. Furthermore, a report from Kortum et al. showed that RasGRP1 KO thymi showed increased ratios of DN/DP and DN3/DN4 compared to wildtype thymi, implying defects in β-selection [25]. Interestingly, it was also recently discovered that Sos1 deficiency enforced within the DN compartment resulted in a partial β-selection block; however, Sos1 deficiency did not impair positive or negative selection [26].

While RasGRP1 KO and RasGRP1/4 double KO mice showed a block in β-selection, an in depth analysis of which β-selection events were RasGRP dependent was not performed. Additionally, since there was a more profound β-selection block in RasGRP1/4 double KO mice, there is evidence that multiple RasGRPs regulate early T cell development and it is possible that RasGRP members other than RasGRP1 and RasGRP4 regulate β-selection. Therefore, we performed a detailed analysis of the hallmark events of β-selection in RasGRP1 KO, RasGRP3 KO and RasGRP1/3 double KO (DKO) mice. Our data indicated that in the absence of RasGRP1 or RasGRP1 and 3, DN thymocytes inefficiently developed into DP thymocytes and showed partial developmental arrest at the DN3 thymocyte stage. Furthermore, RasGRP1 KO and RasGRP1/3 DKO thymi showed impaired early DN3 (DN3E) to late DN3 (DN3L) development and a loss of DN3 proliferation, due to defective β-selection. Interestingly, we found that RasGRP1, but not RasGRP3, was required for ERK activation downstream of CXCR4, which may represent a potential mechanism of RasGRP1 mediated control of the β-selection checkpoint. Our findings demonstrate the importance of RasGRP1 during early thymocyte development and provide a mechanistic link between CXCR4 signaling and β-selection.

## Materials and Methods

### Mice

C57BL/6 mice were purchased from the National Cancer Institute and The Jackson Laboratory. The generation of RasGRP1 KO, RasGRP3 KO and RasGRP1/3 DKO has been previously described [22], [27]. All KO mice were crossed onto the C57BL/6 background. For all strains, mice of both sexes were used between 4 and 16 weeks of age. All mice were treated in accordance with protocols approved by the University of Alberta Animal Care and Use Committee.

### Antibodies and Flow Cytometry

Fluorochrome-conjugated and biotinylated antibodies (Ab) were purchased from eBioscience. Anti-cleaved caspase 3 (Asp-175) and anti-pAKT (pS473) were purchased from Cell Signaling. Anti-pERK (pT202/pY204) was purchased from BD Biosciences. Anti-BrdU (Alexa 647) was purchased from Phoenix Flow Systems. The mCD1d/PBS57 tetramer was kindly provided by the NIH Tetramer Facility. Cells were stained with Ab cocktails in FACS buffer (PBS, 1% FCS, 0.02% sodium azide) for 30 min on ice. Cells were washed twice with FACS buffer following primary and secondary Ab staining. Cells were treated with the BD Fix/Perm kit (BD Biosciences) for intracellular staining of BrdU, cleaved caspase 3 and TCRβ. Cell events were collected on a FACSCanto II (BD Biosciences) and analyzed with FloJo software (Tree Star).

### Cell Sorting

DN3e thymocytes were isolated by FACS using a FACSAria (BD Biosciences) cell sorter. Prior to sorting thymocyte suspensions were enriched for DN thymocytes by magnetic depletion using anti-CD8 and anti-CD4 Abs. DN3e thymocytes were sorted as [CD8/CD3/CD44/CD19]^neg^ and CD25^hi^CD98^lo^.

### OP9-DL1 Culture

OP9-DL1 cells were maintained as previously described [28]. A total of 4×10^4^ DN3E were cultured in DM20 (DMEM, 5 mM Hepes, 50 units (mg/mL) penicillin/streptomycin, 2 mM L-glutamine, 50 mM 2-mercaptoethanol, 50 mg/mL gentamicin sulfate) supplemented with 1 ng/mL IL-7 in 96 wells plates previously seeded with 1.2×10^4^ OP9-DL1 cells at 37°C. Co-cultures were harvested through a 70 µm cell strainer after 24 h and 48 h of culture and analyzed by flow cytometry.

### BrdU

Mice were injected i.p. with 1 mg of BrdU and were euthanized 2 h after injection. Thymocyte BrdU incorporation was assessed by flow cytometry using a protocol adapted from the BD APC BrdU Flow Kit (BD Biosciences). Thymocytes were first stained for surface markers in FACS buffer as described above. Following surface staining, thymocytes were resuspended in BD Cytofix (BD Fix/Perm kit), incubated on ice for 20 min and washed twice in 1X Fix/Perm buffer (BD Fix/Perm kit). Cells were then treated with a solution of 90% FCS and 10% DMSO on ice for 10 min and washed twice in 1X Fix/Perm buffer. Thymocytes were again treated with BD Cytofix on ice for 5 min and washed twice in 1X Fix/Perm buffer. Cells then underwent DNase treatment for 1 h at 37°C and were washed twice in 1X Fix/Perm buffer. Thymocytes were then stained with anti-BrdU in 1X Fix/Perm buffer for 30 min on ice, washed twice in FACS buffer and analyzed by flow cytometry.

### SDF-1α Stimulations

Total thymocytes were resuspended at 1×10^7^ cells/mL in DMEM with 0.1% BSA. Cells were pre-treated or not with 20 µM of the PI3K inhibitor, LY294002, for 30 min at 37°C. Cells were stimulated with 10 nM SDF-1α (PeproTech) for various time points, immediately fixed in 1X BD Phosflow Lyse/Fix Buffer (BD Biosciences) and washed once with FACS buffer. Thymocytes were then permeabilized in 90% methanol on ice for a minimum of 30 min and washed twice in FACS buffer. AKT activation was detected with anti-pAKT (1/100) followed by incubation with an Alexa fluor 647 conjugated anti-rabbit secondary Ab. ERK activation was detected with anti-pERK (1/400) followed by incubation with biotin conjugated anti-mouse Ab and APC conjugated streptavidin. Cells were also stained with Abs to surface markers and analyzed by flow cytometry. ERK/AKT phosphorylation was quantified as fold induction of mean fluorescent intensities of stimulated samples/unstimulated samples.

### Statistics

Mean, SD and P values were calculated using Prism software (Graphpad) using a two-tailed, unpaired Student’s t-test. Asterisks represent statistically significant analyses comparing B6 to RasGRP mutant mice, unless indicated otherwise. Statistical significance is represented as */#p<0.05, **p<0.01 and ***p<0.001.

## Results

Given that the Ras/ERK pathway is activated during β-selection and Sos1 deficiency only partially impaired the DN3 to DN4 transition, it is probable that other RasGEFs are involved in β-selection. Recent reports published by Zhu et al. and Kortum et al. showed that RasGRP1- and RasGRP1/4- deficient mice showed impaired development beyond DN3, suggesting impaired β-selection [24], [25]. However, the involvement of RasGRP family members in the hallmark events of β-selection was not explored in detail.

**Figure 1:**
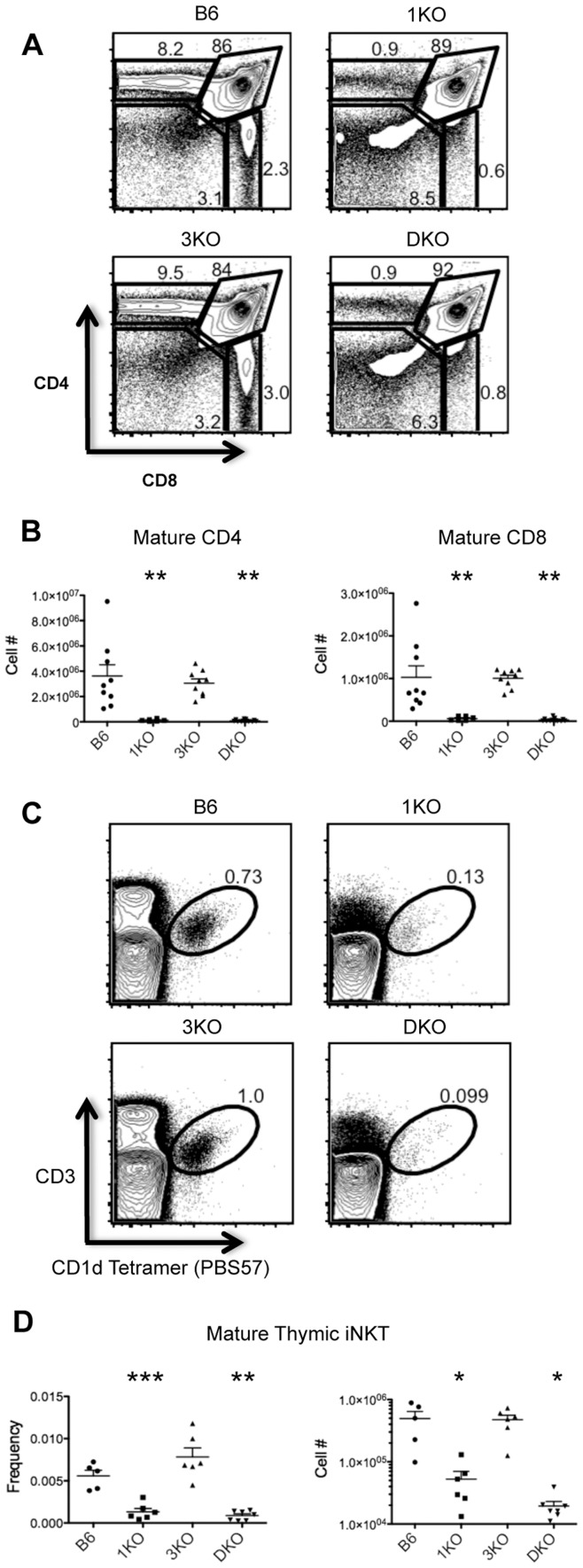
RasGRP1, but not RasGRP3, is required for thymocyte positive selection and iNKT selection. **A.** CD4 by CD8 profiles of Thy1.2^+^ cells from B6 (n = 9), 1KO (n = 6), 3KO (n = 9) and DKO thymi (n = 13). **B.** Numbers of mature Thy1.2^+^CD3^+^CD44^−^ CD4 and CD8 SP thymocytes. **C.** CD3 by CD1d Tetramer (PBS57) profiles of bulk thymocytes from B6 (n = 5), 1KO (n = 6), 3KO (n = 6) and DKO (n = 7) thymi. **D.** Frequencies and numbers of mature CD3^+^CD1d(PBS57) Tetramer^+^ iNKT cells. *p<0.05, **p<0.01 and ***p<0.001.

To examine potential roles for RasGRP1 and/or RasGRP3 in T cell development, we first examined CD4/CD8 profiles of Thy1.2^+^ thymocytes from wildtype (B6), RasGRP1^−/−^ (1KO), RasGRP3^−/−^ (3KO) and RasGRP1^−/−^; 3^−/−^ (DKO) mice. As has been previously reported, 1KO mice showed significantly reduced frequencies and numbers of CD4SP and CD8SP thymocytes (Fig. 1A,B) due to defects in positive selection [22], [29]. Likewise, DKO mice also showed significant reductions in CD4SP and CD8SP frequencies and numbers. However, double deficiency of RasGRP1 and RasGRP3 did not appear to further abrogate positive selection compared to RasGRP1 deficiency alone. 3KO mice did not show significant alterations in numbers or frequencies of any major thymic subsets.

In addition to defects in positive selection, 1KO thymi have recently been reported to show impaired iNKT cell development [23]. To assess a potential additional role for RasGRP3 in thymic iNKT cell development we examined B6 and RasGRP1/3 deficient thymi for the presence of mature CD1d(PBS57) tetramer binding CD3^+^ iNKT cells. As expected, 1KO and DKO thymi showed statistically significant reductions in iNKT cell frequencies and numbers compared to B6 (Fig. 1C,D). 3KO thymi showed similar numbers and frequencies of iNKT cells as B6 and iNKT cell selection did not appear further abrogated by RasGRP1/3 double deficiency compared to RasGRP1 loss alone.

**Figure 2:**
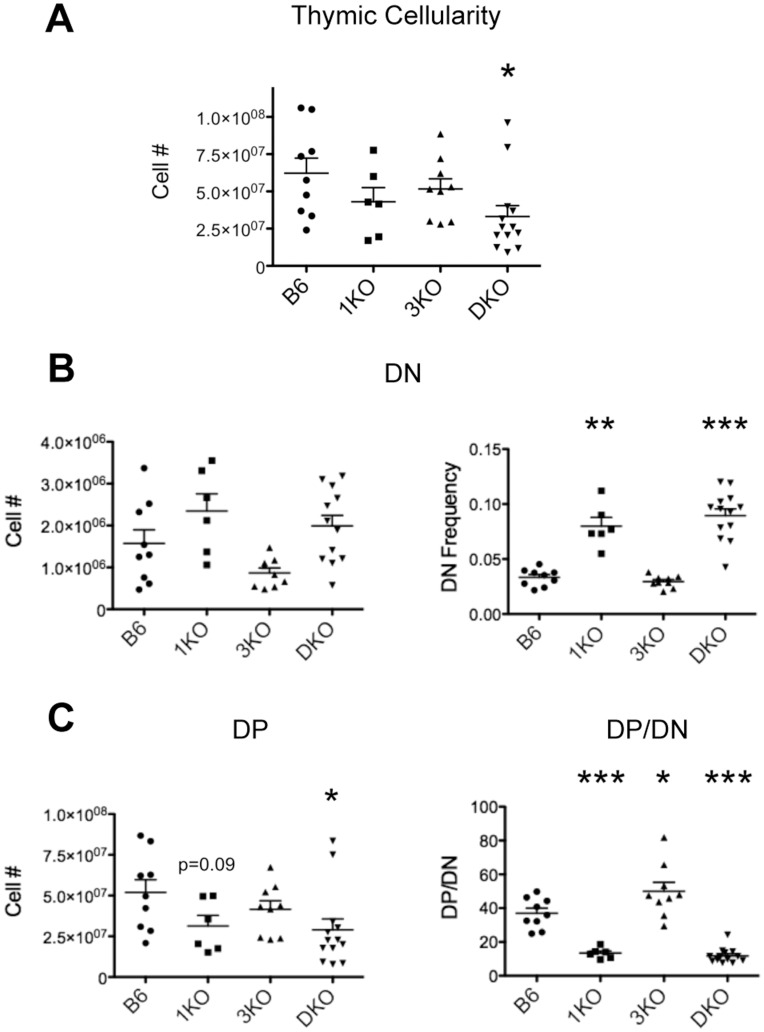
RasGRP1 KO and RasGRP1/3 DKO mice display inefficient generation of DP thymocytes. **A.** Numbers of thymocytes from B6 (n = 9), 1KO (n = 6), 3KO (n = 9) and DKO (n = 13) thymi. **B.** Numbers and frequencies of DN (CD4^−^CD8^−^Thy1.2^+^CD3^lo^) from B6 (n = 9), 1KO (n = 6), 3KO (n = 9) and DKO (n = 13) thymi. **C.** left, numbers of DP (CD4^+^CD8^+^Thy1.2^+^); right, ratio of frequencies of DP/DN. *p<0.05, **p<0.01 and ***p<0.001.

To gain a better understanding of the influence of RasGRP1, RasGRP3 and RasGRP1/3 deficiency on β-selection, we examined total thymic cellularity since the proliferative burst that accompanies β-selection is largely responsible for the total number of thymocytes present. As a result of inefficient β-selection, DKO thymi showed a significant reduction in total thymic cellularity compared to B6. 1KO and 3KO thymi showed a reduction in total thymocyte numbers compared to B6, however, this was not statistically significant (Fig. 2A). An important outcome of β-selection is the development of DP thymocytes from DN progenitors. Likewise, defects in β-selection disrupt the normal balance of DN to DP in the thymus. Interestingly, 1KO and DKO mice showed significantly elevated frequencies and increased, although not statistically significant, numbers of DN thymocytes compared to B6 (Fig. 2B). In addition to having an increased pool of DN, 1KO and DKO thymi also show decreased numbers of DP compared to B6 (Fig. 2C). Since the DP compartment is generated from DN progenitors, analyzing the ratio of DP to DN (DP/DN) provides insight into the efficiency with which DN thymocytes develop into DP. Strikingly, 1KO and DKO mice showed significant reductions in DP/DN, suggesting inefficient developmental progression through the DN stages, resulting in fewer DP (Fig. 2C). In contrast, 3KO mice showed a modest increase in DP/DN, which may represent more efficient development of DP due to predominantly RasGRP1 driven signaling during development. No statistical difference in DP/DN ratio was observed between 1KO and DKO mice. Therefore, it appears that in addition to regulating positive selection, RasGRP1 also regulates the generation of DP.

**Figure 3:**
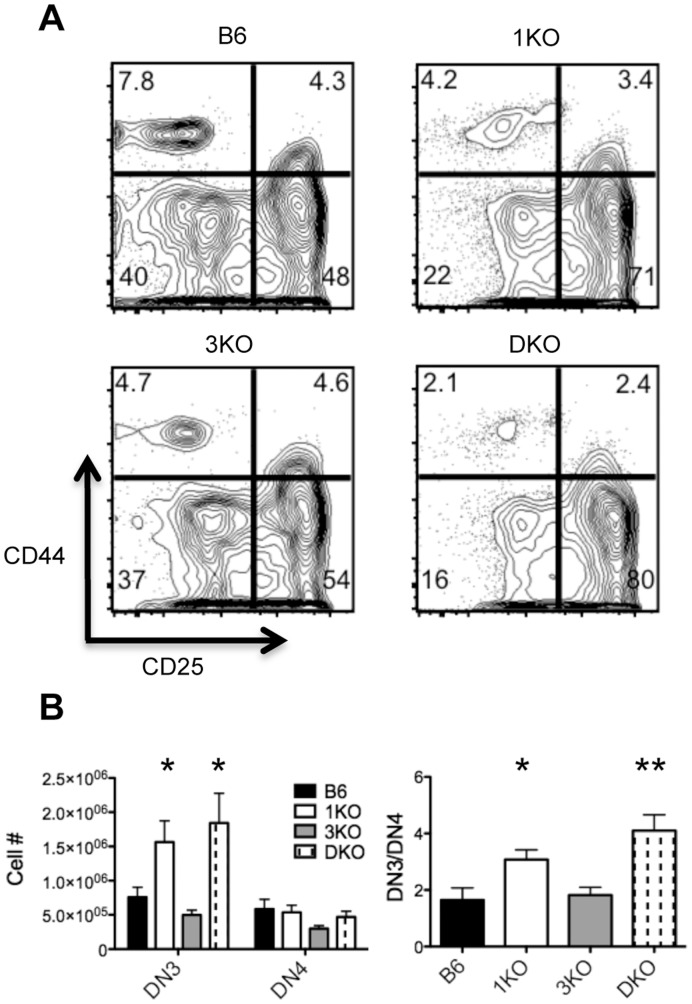
RasGRP1 KO and RasGRP 1/3 DKO show inefficient thymocyte development beyond DN3. **A.** CD44 by CD25 profiles of DN thymocytes (CD4^−^CD8^–^Thy1.2^+^CD3^lo^) from B6 (n = 9), 1KO (n = 6), 3KO (n = 9) and DKO (n = 13) thymi. **B.** left, numbers of DN3 (CD4^−^CD8^−^CD3^lo^CD44^−^CD25^+^) thymocytes and DN4 (CD4^−^CD8^−^CD3^lo^CD44^−^CD25^−^) thymocytes; right, ratio of frequencies of DN3/DN4.

Since RasGRP1 deficiency results in inefficient DN to DP development, we next examined the DN compartment of wildtype and RasGRP1/3 deficient thymi using the CD44/CD25 profile of DN (CD4^−^CD8^−^Thy1.2^+^CD3^lo^) thymocytes. We found that 1KO and DKO thymi showed significantly increased frequencies and numbers of DN3 thymocytes (CD44^−^CD25^+^) relative to B6 (Fig. 3A,B), suggesting defects in β-selection. One important result of β-selection is differentiation of DN3 thymocytes into DN4 thymocytes (CD44^−^CD25^−^). To evaluate this critical differentiation step in the thymocyte developmental program, we examined the ratio of frequencies of DN3 to DN4 thymocytes. 1KO and DKO mice showed significant increases in DN3/DN4, providing further evidence of impaired β-selection in the absence of RasGRPs (Fig. 3B). The DN3/DN4 seemed to be modestly higher in DKO thymi compared to 1KO, although this was not statistically significant. Loss of RasGRP3 alone did not appear to influence the development of DN3 into DN4 and the contribution of RasGRP1 to β-selection appears to be dominant to RasGRP3.

**Figure 4:**
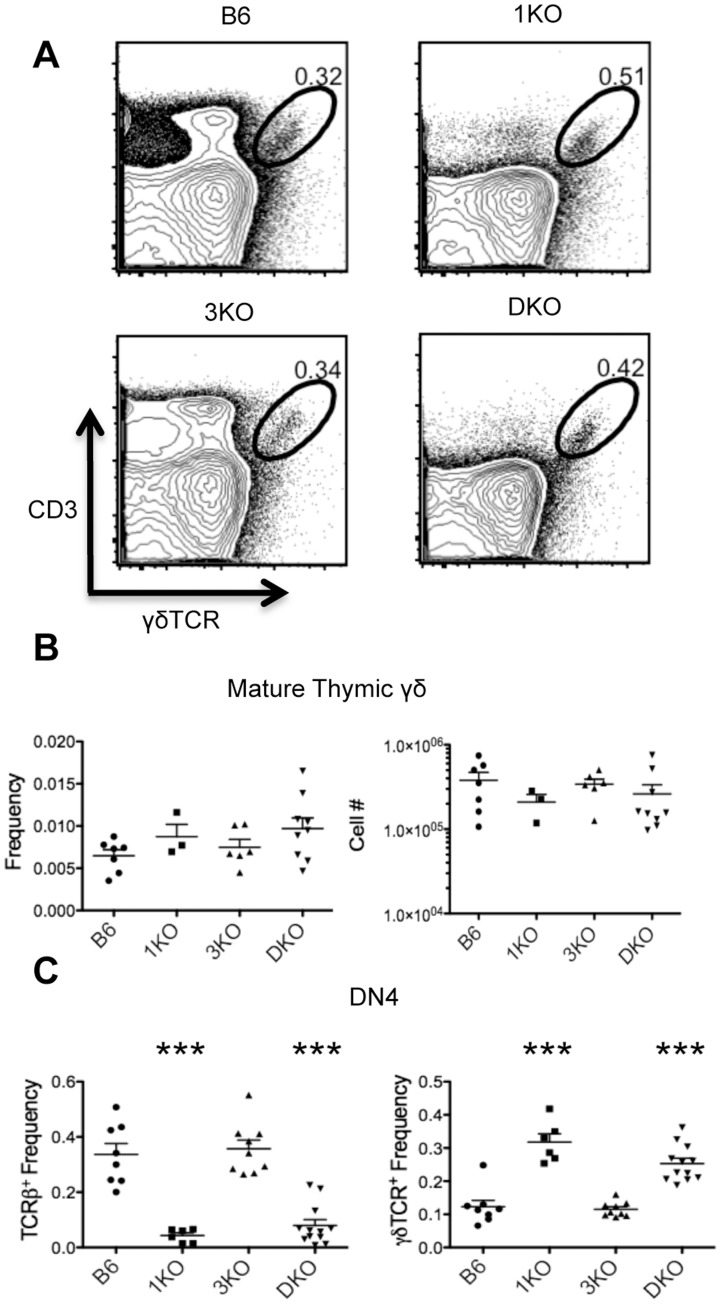
RasGRP1 KO and RasGRP1/3 DKO DN4 thymocytes show an increased frequency of γδ T cells, despite normal numbers and frequencies of mature thymic γδ T cells. A. CD3 by γδTCR profiles of bulk thymocytes from B6 (n = 7), 1KO (n = 3), 3KO (n = 6) and DKO (n = 9) thymi. **B.** Frequencies and numbers of mature CD3^+^γδTCR^+^ γδ T cells. **C.** left, frequencies of surface TCRβ^+^ DN4 (CD4^−^CD8^−^Thy1.2^+^CD44^−^CD25^−^) cells from B6 (n = 8), 1KO (n = 6), 3KO (n = 9) and DKO (n = 12) thymi; right, frequencies of surface γδTCR^+^ DN4 cells. ***p<0.001.

The bifurcation of αβ and γδ T cell development is thought to occur at the DN3 stage. Since we saw a significant increase in DN3 numbers in RasGRP1/3 deficient thymi and DN3 can give rise to both αβ and γδ T cells, we wanted to address possible alterations in γδ T cell development due to RasGRP1/3 deficiency. To examine thymic γδ development we looked for the presence of mature γδTCR^+^CD3^+^ thymocytes in B6 and RasGRP1/3 deficient thymi. We found 1KO, 3KO and DKO thymi showed similar numbers and frequencies of mature γδ thymocytes as B6 (Fig. 4 A,B). These results suggested that the increased DN3/DN4 seen in RasGRP1/3 deficient thymi was likely due to defects in αβ development and not γδ development. Therefore, we next focused on αβ development from DN3 in RasGRP1/3 deficient mice.

Following β-selection, DN4 upregulate surface expression of TCRβ as they mature into DP thymocytes. To gain insight into progression through β-selection, we examined DN4 for surface expression of TCRβ. 1KO and DKO thymi showed statistically significant reductions in frequencies of surface TCRβ^+^ DN4 compared to B6, while 3KO thymi showed similar frequencies of surface TCRβ^+^ DN4 as B6 (Fig. 4C). Since γδ T cells also contribute to the DN4 pool, we examined DN4 for surface expression of γδTCR. 1KO and DKO thymi showed statistically significant increases in frequencies of γδTCR^+^ DN4 compared to B6, while 3KO DN4 showed similar frequencies as B6 (Fig. 4C). Again, no statistical difference between 1KO and 3KO was observed. Although 1KO and DKO thymi showed elevated frequencies of γδTCR^+^ DN4, numbers and frequencies of mature γδ T cells remained similar to B6 (Fig. 4A,B). Altogether these results suggest that the loss of RasGRP1 results in an altered pool of DN4 due to impaired β-selection, however αβ and γδ lineage commitment appears to remain intact.

**Figure 5:**
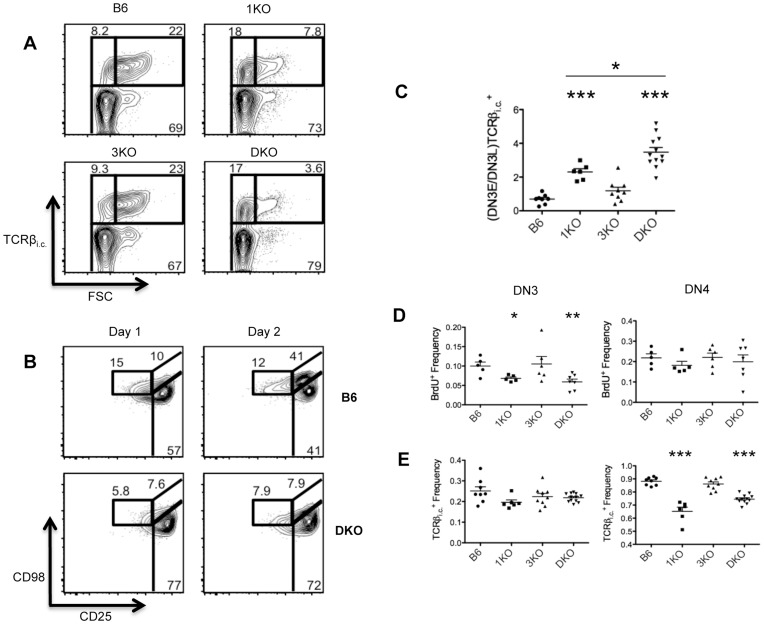
RasGRP1 KO and RasGRP1/3 DKO thymocytes display impaired proliferation of DN3 and inefficient transition from DN3E to DN3L. A. Intracellular TCRβ (TCRβ_i.c._) by forward scatter (FSC) profiles of DN3 (CD4^−^CD8^−^Thy1.2^+^CD44^−^CD25^+^) from B6 (n = 8), 1KO (n = 6), 3KO (n = 9) and DKO (n = 12) thymi. **B.** Frequencies of DN3 (CD4^−^CD8^−^Thy1.2^+^CD44^−^CD25^+^) and DN4 (CD4^−^CD8^−^Thy1.2^+^CD44^−^CD25^−^) expressing intracellular TCRβ (TCRβ_i.c._). **C.** Ratio of frequencies of TCRβ_i.c._^+^ DN3E/DN3L ((DN3E/DN3L)TCRβ_i.c._^+^). **D.** CD98 by CD25 profiles of Thy1.2^+^CD44^−^ cells from 1 and 2 day DN3E-OP9-DL1 co-cultures; data are representative of 3 independent experiments. **E.** Frequencies of BrdU^+^ DN3 (CD4^−^CD8^−^Thy1.2^+^CD44^−^CD25^+^) and DN4 (CD4^−^CD8^−^Thy1.2^+^CD44^–^CD25^−^) from B6 (n = 5), 1KO (n = 5), 3KO (n = 6) and DKO (n = 7) mice injected with BrdU i.p. 2h prior to euthanasia. *p<0.05, **p<0.01 and ***p<0.001.

To evaluate the passage of DN3 through β-selection more closely, we examined early DN3 (DN3E) and late DN3 (DN3L) populations of B6, 1KO, 3KO and DKO thymocytes. DN3E^TCRβi.c.+^ are DN3 that have undergone successful VDJ recombination, but have not yet undergone pre-TCR driven proliferation. In contrast, DN3L^TCRβi.c.+^ are DN3 that have received a pre-TCR signal and as a result undergo blastogenesis, which can be detected as changes in cell size [30], [31]. To focus our analysis on the DN3E to DN3L transition specifically, we examined intracellular TCRβ^+^ (TCRβ^+^_i.c._) DN3 by forward scatter (FSC). The DN3 compartment of 1KO and DKO thymi showed increased frequencies of DN3E^TCRβi.c.+^ and decreased frequencies of DN3L^TCRβi.c.+^ compared to B6 and 3KO DN3 (Fig. 5A), despite showing similar frequencies of total TCRβ_i.c._^+^ DN3 (Fig. 5B), suggesting defective TCRβ rearrangement does not underlie impaired β-selection. Of note, 1KO and DKO thymi showed significantly reduced frequencies of TCRβ_i.c._^+^ DN4 compared to B6 (Fig. 5B), providing further confirmation that the composition of the DN4 compartment is altered in RasGRP1 and RasGRP1/3-deficient thymi. To gain more insight into progression through β-selection, we calculated the ratio of frequencies of DN3E^TCRβi.c.+^ to DN3L^TCRβi.c.+^ over multiple experiments. 1KO and DKO thymi showed a significant increase in (DN3E/DN3L)TCRβ_i.c._^+^ ratios compared to B6 (Fig. 5C), strongly suggesting DN3E are unable to undergo efficient blastogenesis in the absence of RasGRPs. Furthermore, DKO DN3 showed significantly higher (DN3E/DN3L)TCRβ_i.c._^+^ ratios than 1KO mice. Consistent with our previous data, 3KO mice appear to undergo normal β-selection and show similar (DN3E/DN3L)TCRβ_i.c._^+^ ratios as B6.

To obtain further confirmation that RasGRP1/3 deficient thymocytes were impaired in the DN3E to DN3L transition, we tested the ability of B6 and DKO DN3E to mature in the *in vitro* OP9-DL1 model of T cell development. DN3E (CD25^+^CD98^lo^) were isolated from B6 and DKO thymi by FACS, seeded on OP9-DL1 monolayers and Thy1.2^+^CD44^lo^ DN were analyzed after 1 and 2 days of co-culture for the expression of surface markers CD98 and CD25. Consistent with our previous *in vivo* data, DKO DN3E (CD25^+^CD98^lo^) were unable to transition to DN3L (CD25^+^CD98^hi^) after 2 days of co-culture, while B6 DN3E underwent extensive maturation to DN3L (Fig. 5D). Altogether, our *in vivo* and *in vitro* data strongly suggest that RasGRP1 is required for efficient β-selection of DN3E and subsequent differentiation to DN3L. However, we next wanted to address impact of RasGRP1/3 ablation on other aspects of β-selection.

One important result of β-selection is extensive proliferation of DN3s expressing a functionally rearranged TCRβ. The lack of DN3L in RasGRP1/3 deficient mice suggested defects in DN3 proliferation. To address proliferation in RasGRP1/3 deficient thymi more directly, we injected mice with BrdU i.p. 2 hours prior to euthanasia and assayed BrdU incorporation in DN3 and DN4 thymocytes. 1KO and DKO thymi showed a significant decrease in the frequency of BrdU^+^ DN3 compared to B6, but not compared to each other, while 3KO DN3 showed no significant difference in BrdU incorporation compared to B6 (Fig. 5E). There were no significant differences in DN4 BrdU incorporation between any of the mice examined, suggesting DN4 proliferation occurs independently of RasGRP1 activity (Fig. 5E). Altogether, these data suggest that defective β-selection driven proliferation of DN3 is a major consequence of RasGRP1 loss.

**Figure 6:**
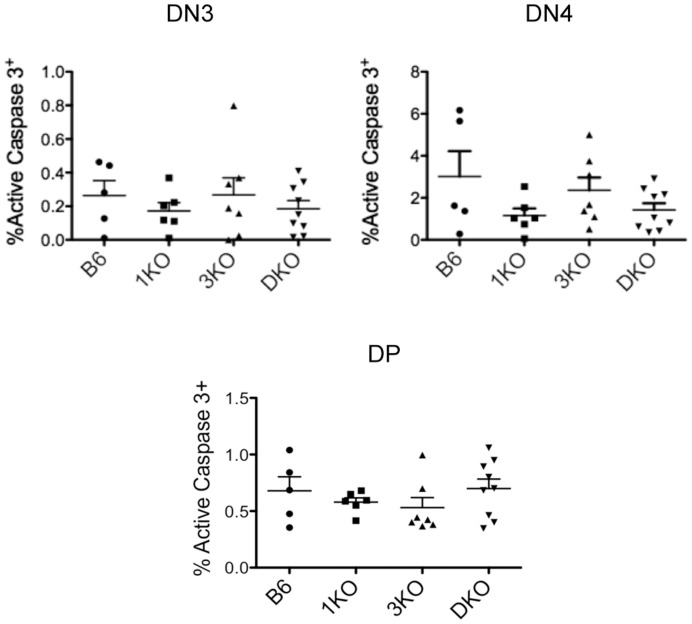
RasGRP1 KO, RasGRP3 KO and RasGRP1/3 DKO thymocytes show intact survival. Percentages of DN3 (CD4^−^CD8^−^Thy1.2^+^CD44^−^CD25^+^), DN4 (CD4^−^CD8^−^Thy1.2^+^CD44^−^CD25^−^) and DP (CD4^+^CD8^+^Thy1.2^+^) showing active caspase 3.

Another important result of β-selection is the survival of developing thymocytes as they differentiate from DN3 to DN4. Therefore, changes in the apoptotic activity of developing DN3/DN4 may result in changes in β-selection. To address thymocyte apoptosis, we examined caspase 3 activation in DN3, DN4 and DP thymocyte subsets from B6, 1KO, 3KO and DKO mice. We found that 1KO, 3KO and DKO thymi showed no significant differences in the percentages of active caspase 3^+^ DN3, DN4 or DP compared to B6 (Fig. 6). In fact, it appears that defective β-selection in 1KO and DKO thymi mildly reduced the frequency of active caspase 3^+^ DN3 and DN4, suggesting that RasGRP1/3 deficiency does not impair thymocyte survival. Furthermore, normal frequencies of active caspase 3^+^ DP thymocytes from RasGRP1/3 deficient thymi suggest that reduced DP/DN ratios observed in RasGRP1/3 deficient thymi are a result of inefficient DN to DP development, rather than increased DP apoptosis.

**Figure 7:**
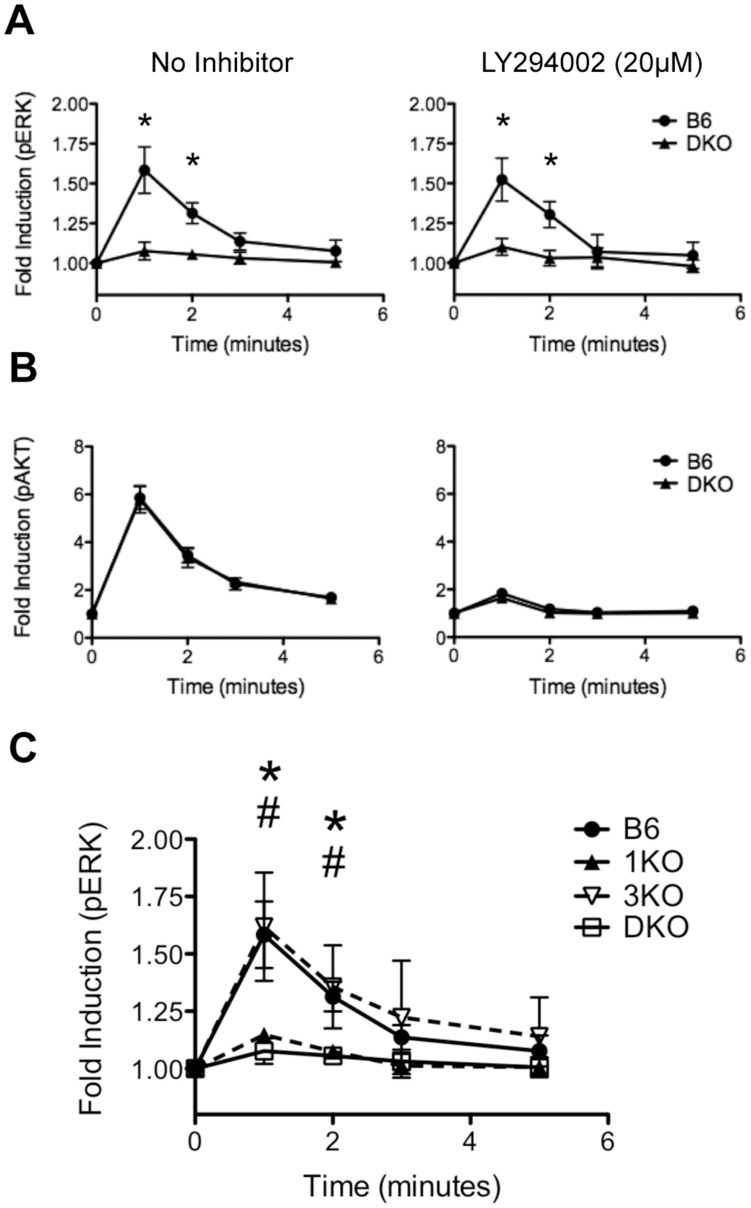
DN3 require RasGRP1 for ERK phosphorylation in response to CXCR4 activation. **A, B.** Bulk thymocytes were pre-treated with (right panels) or without (left panels) 20 µM of the PI3K inhibitor LY294002 and were stimulated with 10 nM SDF1α for 0, 1, 2, 3 or 5 minutes. **A.** Induction of p-ERK (pT202/pY204) was measured in DN3 (CD4^−^CD8^−^Thy1.2^+^CD3^lo^ Thy1.2^+^CD44^−^CD25^+^) from B6 and DKO thymi. **B.** Induction of p-AKT (pS473) was measured in DN3 (CD4^−^CD8^−^Thy1.2^+^CD3^lo^ Thy1.2^+^CD44^−^CD25^+^) from B6 and DKO thymi. **C.** Bulk thymocytes were stimulated with 10 nM SDF1α for 0, 1, 2, 3 or 5 minutes and the induction of p-ERK (pT202/pY204) was measured in DN3 (CD4^−^CD8^−^Thy1.2^+^CD3^lo^ Thy1.2^+^CD44^−^CD25^+^) from B6, 1KO, 3KO and DKO thymi. Data are representative of between 3 and 7 independent experiments. * represents a significant statistical comparison between B6 and DKO. # represents a significant statistical comparison between B6 and 1KO. */# p<0.05.

Signaling through CXCR4 has recently been shown to be an important co-stimulator of pre-TCR signaling and is required for efficient β-selection [12]. Stimulating DN3 through CXCR4 with SDF1α results in the phosphorylation and activation of ERK and AKT, two kinases that are thought to be important during β-selection. To address whether RasGRP1/3 are involved in CXCR4-dependent ERK and AKT activation, we stimulated B6 and DKO thymocytes with 10 nM SDF1α and examined the phosphorylation of ERK and AKT in DN3. DKO DN3 showed an almost complete loss of ERK phosphorylation after 1 min and 2 min of SDF1α stimulation compared to B6 (Fig. 7A). In contrast, B6 and DKO DN3 showed similar fold inductions of AKT activation at all points (Fig. 7B). These results suggest that RasGRP1/3 are required for ERK activation in response to SDF1α/CXCR4 signaling, but RasGRP1/3 deficiency does not impair PI3K/AKT signaling in response to SDF1α. Interestingly, RasGRP1 contains a C-terminal plasma membrane targeting (PT) domain that includes a basic/hydrophobic cluster of amino acids shown to interact with phosphoinositides [32], [33]. Therefore, targeting of RasGRP1/3 to the plasma membrane and subsequent Ras activation may be dependent on PI3K activity. To address whether PI3K may be regulating RasGRP1/3 during CXCR4 signaling, we carried out the same stimulations in presence of 20 µM of the PI3K inhibibitor, LY294002. We found that inhibition of PI3K had little to no effect on ERK activation in B6 or DKO DN3 (Fig. 7A). LY294002 treated DN3 from B6 and DKO thymi showed similar fold inductions of pERK in the presence or absence of inhibitor and B6 DN3 maintained significantly higher fold inductions of pERK compared to DKO. Also, as a negative control we measured pAKT fold induction in LY294002 treated DN3 and saw a dramatic loss of AKT activation in both mice as expected (Fig. 7B). Therefore, although RasGRP1/3 are required for ERK activation in response to SDF1α, PI3K activity does not regulate RasGRP1/3 mediated ERK activation during CXCR4 signaling.

Although RasGRP1/3 deficient DN3 were unable to activate ERK in response to CXCR4 signaling, analysis of DKO DN3 could not distinguish the individual contributions of RasGRP1 and RasGRP3 to ERK activation downstream of CXCR4. To gain insight into the individual roles of RasGRP1 and RasGRP3 during CXCR4 mediated ERK activation, we carried out the previously described SDF1α stimulations using 1KO and 3KO DN3 (Fig. 7C). 3KO DN3 showed similar fold inductions of pERK as B6 in response to SDF1α stimulation at all time points. In contrast, 1KO and DKO DN3 were equally impaired in their ability to activate ERK following SDF1α stimulation. Therefore, RasGRP1, but not RasGRP3, is required for ERK activation downstream of CXCR4.

## Discussion

Using a comprehensive analysis of early thymocyte development, we were able to elucidate the effects of RasGRP1, RasGRP3 and RasGRP1/3-deficiency on thymocyte β-selection. 1KO and DKO displayed impaired differentiation and proliferation of DN3 thymocytes as they transition from DN3E to DN3L, despite intact TCRβ expression. Additionally, the DN to DP transition in 1KO and DKO mice was reduced. Of note, we found that despite showing elevated frequencies of DN4 γδ T cells, RasGRP1 and/or RasGRP3 does not appear to regulate αβ vs γδ lineage commitment. Finally, we found that 1KO and DKO DN3 thymocytes were defective in ERK activation following SDF1α stimulation, which may contribute to impaired β-selection. Our findings provide a basis for understanding RasGRP mediated control of the β-selection checkpoint and the downstream consequences of inefficient RasGRP-mediated Ras activation during thymopoiesis.

In most cases, RasGRP1 and RasGRP1/3-deficient thymocytes displayed equivalent deficiencies in β-selection, while 3KO mice were mostly normal. Therefore, we attribute most of the deficiencies in β-selection observed in DKO mice to a loss of RasGRP1 and suggest that RasGRP3 cannot compensate for the loss of RasGRP1. Indeed, it has been shown that RasGRP1 is the most highly expressed RasGRP member in DN3a thymocytes [34]. The lack of a difference between RasGRP1 KO and RasGRP1/3 DKO mice contrasts the finding of the Zhang group where RasGRP4-defient mice showed no impairment in β-selection, but the combined loss of RasGRP1 and 4 showed a more profound phenotype than RasGRP1 deficiency alone. This suggests that RasGRP4 could compensate somewhat for the loss of RasGRP1 [24]. The difference observed between RasGRP1/3 DKO and RasGRP1/4 DKO is likely due to relatively higher expression of RasGRP4 than RasGRP3 in DN3 thymocytes as reported by the Immunological Genome Project [24], [34].

The development of DN into DP is a complex multi-stage program involving interactions between developing thymocytes and the diverse elements that make up the thymic microenvironment. RasGRP1 ablation results in inefficient development of DN into DP (Fig. 2b). Signaling downstream of the pre-TCR is known to involve the signaling molecules Zap70, Syk, LAT and SLP76, as well as activation of the Ras/ERK signaling pathway [5]–[10]. Given that RasGRP1 contains a physiologically relevant C1 domain that binds DAG, it is likely that LAT mediated PLCγ recruitment, activation and subsequent DAG production in response to pre-TCR signaling recruits RasGRP1 to the plasma membrane, resulting in Ras activation [2], [35]. In support of this mode of RasGRP1 regulation, although not extensively studied, mice with a LAT Y136F mutation that abrogates PLCγ recruitment and activation show impaired DN to DP development, suggesting impaired β-selection [36], [37]. However, RasGRP1 regulation downstream of the pre-TCR remains poorly understood.

We have identified a novel role for RasGRP1 downstream of CXCR4 activation in DN3 thymocytes. RasGRP1 deficient DN3 cells are unable to activate ERK in response to SDF1α stimulation of CXCR4. However, RasGRP1 deficient DN3 are able to activate AKT downstream of CXCR4 activation. Interestingly, CXCR4 deficient thymi show impaired β-selection and signals transduced through CXCR4 are important during early T cell development [12]. The mechanism of RasGRP1 activation downstream of CXCR4 remains unclear. To address one possible mechanism of RasGRP1 activation in response to CXCR4 signaling we carried out SDF1α stimulations of DN3 thymocytes in the presence of PI3K inhibitor, LY294002. RasGRP1 contains a C-terminal PT domain that has been shown to bind phosphoinositides and recruit RasGRP1 to the plasma membrane [32], [33]. However, we found that inhibition of PI3K activity had no effect on ERK activation in response to SDF1α stimulation, suggesting PI3K mediated phoshoinositide generation is not required for RasGRP1 mediated ERK activation downstream of CXCR4. Both RasGRP1 and RasGRP3 contain DAG binding C1 domains that have been classically thought to recruit RasGRPs to Ras containing membranes [18]. Therefore, it appears likely that the mechanism of RasGRP1 activation downstream of CXCR4 is PLCγ dependent and involves RasGRP1 binding membrane DAG through its C1 domain. Interestingly, ERK activation downstream of CXCR4 is also defective in RAG2^−/−^ DN3, unable to express a pre-TCR [38]. Since CXCR4 mediated ERK activation is pre-TCR dependent, RasGRP1 may activate ERK downstream of the pre-TCR. It is known that LAT is part of the pre-TCR signaling complex and that LAT can recruit and activate PLCγ [39]. Given the potential for PLCγ mediated DAG production downstream of the pre-TCR, we predict that RasGRP1 activation during β-selection is PLCγ dependent. However, more experiments are needed to address the precise mechanism of RasGRP1/3 activation in thymocytes.

The TCR signaling strength model of early T cell development suggests that the strength of the TCR signal transduced in DN3E thymocytes is the deciding determinant of αβ vs γδ lineage choice [40]. It is thought that strong signals transduced through the γδTCR lead to strong ERK activation and increased expression of EGRs and Id3, driving γδ T cell selection [41]. In contrast, weak signals transduced through the pre-TCR, resulting in weak ERK activation, in conjunction with Notch and CXCR4 signals promote β-selection [4]. Interestingly, targeted Sos1 KO thymi show similar frequencies, but reduced numbers, of mature γδ T cells compared to wild type thymi [26]. We found that RasGRP1/3 deficiency had no significant effect on frequencies or numbers of mature γδ T cells. A recent report from Chen et al. confirmed that RasGRP1 deficient thymi show uninterrupted γδ T cell development, further suggesting that RasGRP1 is dispensable for thymic γδ T cell development [42]. Interestingly, the DN4 compartment of RasGRP1 deficient thymi shows an increased frequency of γδTCR expressing cells and a paucity of TCRβ expressing cells. Since the numbers and frequencies of mature γδ T cells are similar to wildtype in RasGRP1/3 deficient thymi, the loss of TCRβ expressing DN4 and increase in γδTCR expressing DN4 in RasGRP1 deficient thymi is likely the result of inefficient β-selection, rather than altered lineage commitment. Chen et al. also saw increased frequencies of subsets of γδ T cells in RasGRP1 deficient thymi, however normal overall numbers of thymic γδ T cells suggest development remains intact in the absence of RasGRP1 [42]. Therefore, it appears that neither Sos1 nor RasGRP1 act alone in transducing signals required for γδ T cell selection. However, there is evidence suggesting Sos1 and RasGRP1 cooperate in a feedforward loop to coordinate Ras/ERK activation [20], [21]. Structural analysis of Sos1 has revealed an allosteric Ras-GTP binding pocket, which may enhance Sos1 RasGEF activity upon initial Ras activation by RasGRP1. Examination of γδ T cell development in the context of RasGRP1 and Sos1 double deficiency may shed light onto the physiological relevance of this cooperative model of Ras activation and the involvement of these different classes of RasGEFs during γδ T lymphopoiesis.

Although RasGRP1 deficient and targeted Sos1 KO thymi show similar γδ and αβ early T cell development phenotypes, there are some subtle, yet important, differences in the β-selection phenotypes of these different mouse models. Deceptively, both RasGRP1 deficient and Sos1 KO thymi show significantly elevated DN3/DN4 ratios [26]. However, Sos1 KO thymi show a significant reduction in DN4 numbers accompanied by modestly reduced DN3 numbers, while RasGRP1/3 deficient thymi show significantly elevated DN3 numbers and mildly reduced DN4 numbers. Therefore, it appears that the Sos1 KO phenotype lies within the DN4 population rather than within the DN3 population. Furthermore, RasGRP1-deficient and Sos1 KO DN3 and DN4 show opposing proliferation phenotypes. RasGRP1 deficient DN3 show significantly reduced proliferation, while Sos1 KO DN3 cells proliferate normally. In contrast, Sos1KO DN4 show significantly reduced proliferation, while RasGRP1 deficient DN4 show very modest reductions in proliferation. Therefore, Sos1 seems to control the proliferation of DN4 and as a result Sos1 deficiency results in significantly reduced DN4 numbers. It should be noted, complicating this interpretation is the fact that the BrdU pulse times varied between the two studies and this difference may account for the observed differences in proliferation. However, RasGRP1 deficient DN3 show inefficient differentiation from the DN3E to DN3L stages, resulting in significantly elevated DN3 numbers. Interestingly, RasGRP1 deficient thymi show increased DN3 numbers despite significant reductions in DN3 proliferation. This finding clearly demonstrates that increased DN3 cell numbers in RasGRP1 thymi are due to developmental arrest and highlights the intimate connection between DN3 proliferation and their subsequent differentiation into DN4. Altogether these findings suggest that RasGRP1 and Sos1 may regulate temporally distinct events in αβ T cell development. As a result, a feedforward loop involving RasGRP1 mediated Ras activation potentiating Sos1 activity likely does not contribute to β-selection. In support of this, RasGRP1; Sos1 DKO mice show more severely impaired early T cell development phenotypes than either single KO alone, suggesting that Sos1 and RasGRP1 regulate Ras activation independently during T cell development [25]. But, it remains possible that RasGRP1 and Sos1 cooperate in a feedforward loop to regulate γδ T cell development. Interestingly, c-Myc deficient thymocytes show a loss of DN4 proliferation and inefficient DN to DP development [43]. It is known that ERK can phosphorylate c-Myc on Ser62, which stabilizes c-Myc and promotes its activity [44]. Regulation of Ras activation by Sos1 and/or RasGRPs may modulate c-Myc activity to control early thymic T cell development. However, Ras activation has numerous downstream consequences and c-Myc regulation is only one outcome of Ras signaling.

In addition to its classical role as a RasGEF, Sos1 has also been shown to be a Rac activator [45], [46]. Similar to Dbl family members, Sos1 contains tandem Dbl homology (DH) and pleckstrin homology (PH) domains, which control the localization and RacGEF activity of Sos1. Interestingly, Rac1/2 deficient thymocytes show impaired early T cell development, including β-selection [47], [48]. Therefore, it is possible that Sos1 may regulate Rac activation directly during early T cell development, which may account for the different developmental phenotypes of RasGRP1 deficient and Sos1 deficient thymi. However, there is no evidence of Sos1 regulating Rac activation during T cell development and further studies are required to address this possibility.

In conclusion, we showed that RasGRP1 controls the differentiation of DN3E into DN3L and the proliferation of the DN3 compartment. Additionally, RasGRP1 was required for the activation of ERK, downstream of CXCR4. It has become clear that control of the Ras pathway is an important facet of early thymic T cell development. However, many questions remained unanswered. The precise mechanism of RasGRP1 activation during pre-TCR signaling and signaling downstream of CXCR4 remains a mystery. Most importantly, the downstream consequences of Ras activation are poorly understood in the context of T cell development. Much work has been done linking Ras/MAPK signaling to T cell development and has provided invaluable insight into understanding developmental signaling in thymocytes. However, Ras regulates a diverse array of pathways and uncovering the network of Ras regulated signaling pathways in T cells will shed light onto the precise mechanisms by which Ras regulates T cell development.

## References

[1] Bhandoola A, von Boehmer H, Petrie HT, Zuniga-Pflucker JC (2007) Commitment and developmental potential of extrathymic and intrathymic T cell precursors: plenty to choose from. Immunity 26: 678–689. 17582341 10.1016/j.immuni.2007.05.009

[2] Michie AM, Zuniga-Pflucker JC (2002) Regulation of thymocyte differentiation: pre-TCR signals and beta-selection. Semin Immunol 14: 311–323. 12220932 10.1016/s1044-5323(02)00064-7

[3] Hogquist KA, Baldwin TA, Jameson SC (2005) Central tolerance: learning self-control in the thymus. Nat Rev Immunol 5: 772–782. 16200080 10.1038/nri1707

[4] Janas ML, Turner M (2010) Stromal cell-derived factor 1alpha and CXCR4: newly defined requirements for efficient thymic beta-selection. Trends Immunol 31: 370–376. 20829112 10.1016/j.it.2010.07.002

[5] Clements JL, Yang B, Ross-Barta SE, Eliason SL, Hrstka RF, et al (1998) Requirement for the leukocyte-specific adapter protein SLP-76 for normal T cell development. Science 281: 416–419. 9665885 10.1126/science.281.5375.416

[6] Pivniouk V, Tsitsikov E, Swinton P, Rathbun G, Alt FW, et al (1998) Impaired viability and profound block in thymocyte development in mice lacking the adaptor protein SLP-76. Cell 94: 229–238. 9695951 10.1016/s0092-8674(00)81422-1

[7] Sugawara T, Di Bartolo V, Miyazaki T, Nakauchi H, Acuto O, et al (1998) An improved retroviral gene transfer technique demonstrates inhibition of CD4-CD8- thymocyte development by kinase-inactive ZAP-70. J Immunol 161: 2888–2894. 9743350

[8] Zhang W, Sommers CL, Burshtyn DN, Stebbins CC, DeJarnette JB, et al (1999) Essential role of LAT in T cell development. Immunity 10: 323–332. 10204488 10.1016/s1074-7613(00)80032-1

[9] Cheng AM, Negishi I, Anderson SJ, Chan AC, Bolen J, et al (1997) The Syk and ZAP-70 SH2-containing tyrosine kinases are implicated in pre-T cell receptor signaling. Proc Natl Acad Sci U S A 94: 9797–9801. 9275205 10.1073/pnas.94.18.9797 PMC23271

[10] Michie AM, Trop S, Wiest DL, Zuniga-Pflucker JC (1999) Extracellular signal-regulated kinase (ERK) activation by the pre-T cell receptor in developing thymocytes in vivo. J Exp Med 190: 1647–1656. 10587355 10.1084/jem.190.11.1647 PMC2195734

[11] Thompson PK, Zuniga-Pflucker JC (2011) On becoming a T cell, a convergence of factors kick it up a Notch along the way. Semin Immunol 23: 350–359. 21981947 10.1016/j.smim.2011.08.007

[12] Trampont PC, Tosello-Trampont AC, Shen Y, Duley AK, Sutherland AE, et al (2010) CXCR4 acts as a costimulator during thymic beta-selection. Nat Immunol 11: 162–170. 20010845 10.1038/ni.1830 PMC2808461

[13] Janas ML, Varano G, Gudmundsson K, Noda M, Nagasawa T, et al (2010) Thymic development beyond beta-selection requires phosphatidylinositol 3-kinase activation by CXCR4. J Exp Med 207: 247–261. 20038597 10.1084/jem.20091430 PMC2812547

[14] Rincon M, Flavell RA, Davis RJ (2001) Signal transduction by MAP kinases in T lymphocytes. Oncogene 20: 2490–2497. 11402343 10.1038/sj.onc.1204382

[15] Roose JP, Mollenauer M, Gupta VA, Stone J, Weiss A (2005) A diacylglycerol-protein kinase C-RasGRP1 pathway directs Ras activation upon antigen receptor stimulation of T cells. Mol Cell Biol 25: 4426–4441. 15899849 10.1128/MCB.25.11.4426-4441.2005 PMC1140631

[16] Zheng Y, Liu H, Coughlin J, Zheng J, Li L, et al (2005) Phosphorylation of RasGRP3 on threonine 133 provides a mechanistic link between PKC and Ras signaling systems in B cells. Blood 105: 3648–3654. 15657177 10.1182/blood-2004-10-3916

[17] Limnander A, Depeille P, Freedman TS, Liou J, Leitges M, et al (2011) STIM1, PKC-delta and RasGRP set a threshold for proapoptotic Erk signaling during B cell development. Nat Immunol 12: 425–433. 21441934 10.1038/ni.2016 PMC3623929

[18] Johnson JE, Goulding RE, Ding Z, Partovi A, Anthony KV, et al (2007) Differential membrane binding and diacylglycerol recognition by C1 domains of RasGRPs. Biochem J 406: 223–236. 17523924 10.1042/BJ20070294 PMC1948961

[19] Gureasko J, Galush WJ, Boykevisch S, Sondermann H, Bar-Sagi D, et al (2008) Membrane-dependent signal integration by the Ras activator Son of sevenless. Nat Struct Mol Biol 15: 452–461. 18454158 10.1038/nsmb.1418 PMC2440660

[20] Das J, Ho M, Zikherman J, Govern C, Yang M, et al (2009) Digital signaling and hysteresis characterize ras activation in lymphoid cells. Cell 136: 337–351. 19167334 10.1016/j.cell.2008.11.051 PMC2662698

[21] Roose JP, Mollenauer M, Ho M, Kurosaki T, Weiss A (2007) Unusual interplay of two types of Ras activators, RasGRP and SOS, establishes sensitive and robust Ras activation in lymphocytes. Mol Cell Biol 27: 2732–2745. 17283063 10.1128/MCB.01882-06 PMC1899892

[22] Dower NA, Stang SL, Bottorff DA, Ebinu JO, Dickie P, et al (2000) RasGRP is essential for mouse thymocyte differentiation and TCR signaling. Nat Immunol 1: 317–321. 11017103 10.1038/79766

[23] Shen S, Chen Y, Gorentla BK, Lu J, Stone JC, et al (2011) Critical roles of RasGRP1 for invariant NKT cell development. J Immunol 187: 4467–4473. 21957144 10.4049/jimmunol.1003798 PMC3212869

[24] Zhu M, Fuller DM, Zhang W (2012) The role of Ras guanine nucleotide releasing protein 4 in Fc epsilonRI-mediated signaling, mast cell function, and T cell development. J Biol Chem 287: 8135–8143. 22262848 10.1074/jbc.M111.320580 PMC3318739

[25] Kortum RL, Sommers CL, Pinski JM, Alexander CP, Merrill RK, et al (2012) Deconstructing ras signaling in the thymus. Mol Cell Biol 32: 2748–2759. 22586275 10.1128/MCB.00317-12 PMC3416180

[26] Kortum RL, Sommers CL, Alexander CP, Pinski JM, Li W, et al (2011) Targeted Sos1 deletion reveals its critical role in early T-cell development. Proc Natl Acad Sci U S A 108: 12407–12412. 21746917 10.1073/pnas.1104295108 PMC3145744

[27] Coughlin JJ, Stang SL, Dower NA, Stone JC (2005) RasGRP1 and RasGRP3 regulate B cell proliferation by facilitating B cell receptor-Ras signaling. J Immunol 175: 7179–7184. 16301621 10.4049/jimmunol.175.11.7179

[28] Holmes R, Zuniga-Pflucker JC (2009) The OP9-DL1 system: generation of T-lymphocytes from embryonic or hematopoietic stem cells in vitro. Cold Spring Harb Protoc 2009: pdb prot5156. 10.1101/pdb.prot5156 20147086

[29] Priatel JJ, Teh SJ, Dower NA, Stone JC, Teh HS (2002) RasGRP1 transduces low-grade TCR signals which are critical for T cell development, homeostasis, and differentiation. Immunity 17: 617–627. 12433368 10.1016/s1074-7613(02)00451-x

[30] Hoffman ES, Passoni L, Crompton T, Leu TM, Schatz DG, et al (1996) Productive T-cell receptor beta-chain gene rearrangement: coincident regulation of cell cycle and clonality during development in vivo. Genes Dev 10: 948–962. 8608942 10.1101/gad.10.8.948

[31] Taghon T, Yui MA, Pant R, Diamond RA, Rothenberg EV (2006) Developmental and molecular characterization of emerging beta- and gammadelta-selected pre-T cells in the adult mouse thymus. Immunity 24: 53–64. 16413923 10.1016/j.immuni.2005.11.012

[32] Beaulieu N, Zahedi B, Goulding RE, Tazmini G, Anthony KV, et al (2007) Regulation of RasGRP1 by B cell antigen receptor requires cooperativity between three domains controlling translocation to the plasma membrane. Mol Biol Cell 18: 3156–3168. 17567957 10.1091/mbc.E06-10-0932 PMC1949348

[33] Zahedi B, Goo HJ, Beaulieu N, Tazmini G, Kay RJ, et al (2011) Phosphoinositide 3-kinase regulates plasma membrane targeting of the Ras-specific exchange factor RasGRP1. J Biol Chem 286: 12712–12723. 21285350 10.1074/jbc.M110.189605 PMC3069471

[34] Heng TS, Painter MW (2008) The Immunological Genome Project: networks of gene expression in immune cells. Nat Immunol 9: 1091–1094. 18800157 10.1038/ni1008-1091

[35] Bivona TG, Perez De Castro I, Ahearn IM, Grana TM, Chiu VK, et al (2003) Phospholipase Cgamma activates Ras on the Golgi apparatus by means of RasGRP1. Nature 424: 694–698. 12845332 10.1038/nature01806

[36] Sommers CL, Lee J, Steiner KL, Gurson JM, Depersis CL, et al (2005) Mutation of the phospholipase C-gamma1-binding site of LAT affects both positive and negative thymocyte selection. J Exp Med 201: 1125–1134. 15795236 10.1084/jem.20041869 PMC1538971

[37] Fuller DM, Zhu M, Koonpaew S, Nelson MI, Zhang W (2012) The importance of the erk pathway in the development of linker for activation of T cells-mediated autoimmunity. J Immunol 189: 4005–4013. 22984075 10.4049/jimmunol.1201380 PMC3548449

[38] Janas ML, Turner M (2011) Interaction of Ras with p110gamma is required for thymic beta-selection in the mouse. J Immunol 187: 4667–4675. 21930962 10.4049/jimmunol.1101949 PMC3198841

[39] Balagopalan L, Coussens NP, Sherman E, Samelson LE, Sommers CL (2010) The LAT story: a tale of cooperativity, coordination, and choreography. Cold Spring Harb Perspect Biol 2: a005512. 20610546 10.1101/cshperspect.a005512 PMC2908767

[40] Ciofani M, Zuniga-Pflucker JC (2010) Determining gammadelta versus alphass T cell development. Nat Rev Immunol 10: 657–663. 20725107 10.1038/nri2820

[41] Haks MC, Lefebvre JM, Lauritsen JP, Carleton M, Rhodes M, et al (2005) Attenuation of gammadeltaTCR signaling efficiently diverts thymocytes to the alphabeta lineage. Immunity 22: 595–606. 15894277 10.1016/j.immuni.2005.04.003

[42] Chen Y, Ci X, Gorentla B, Sullivan SA, Stone JC, et al (2012) Differential Requirement of RasGRP1 for gammadelta T Cell Development and Activation. J Immunol 189: 61–71. 22623331 10.4049/jimmunol.1103272 PMC3382004

[43] Dose M, Khan I, Guo Z, Kovalovsky D, Krueger A, et al (2006) c-Myc mediates pre-TCR-induced proliferation but not developmental progression. Blood 108: 2669–2677. 16788099 10.1182/blood-2006-02-005900 PMC1895574

[44] Lee T, Yao G, Nevins J, You L (2008) Sensing and integration of Erk and PI3K signals by Myc. PLoS Comput Biol 4: e1000013. 18463697 10.1371/journal.pcbi.1000013 PMC2265471

[45] Innocenti M, Tenca P, Frittoli E, Faretta M, Tocchetti A, et al (2002) Mechanisms through which Sos-1 coordinates the activation of Ras and Rac. J Cell Biol 156: 125–136. 11777939 10.1083/jcb.200108035 PMC2173577

[46] Nimnual AS, Yatsula BA, Bar-Sagi D (1998) Coupling of Ras and Rac guanosine triphosphatases through the Ras exchanger Sos. Science 279: 560–563. 9438849 10.1126/science.279.5350.560

[47] Dumont C, Corsoni-Tadrzak A, Ruf S, de Boer J, Williams A, et al (2009) Rac GTPases play critical roles in early T-cell development. Blood 113: 3990–3998. 19088377 10.1182/blood-2008-09-181180 PMC2673125

[48] Gomez M, Tybulewicz V, Cantrell DA (2000) Control of pre-T cell proliferation and differentiation by the GTPase Rac-I. Nat Immunol 1: 348–352. 11017108 10.1038/79808

